# Comparison of Success Rate and Complications of Totally Percutaneous Decannulation in Patients With Veno-Arterial Extracorporeal Membrane Oxygenation and Endovascular Aneurysm Repair

**DOI:** 10.3389/fmed.2021.724427

**Published:** 2021-08-20

**Authors:** Zhenjie Liu, Yongshan Xu, Xin Xu, Minzhi He, Pan Han, Changming Shao, Yifeng Pan, Libin Zhang, Li Yin, Zhenhua Li, Man Huang, Bing Chen

**Affiliations:** ^1^Department of Vascular Surgery, The Second Affiliated Hospital of Zhejiang University School of Medicine, Hangzhou, China; ^2^Intensive Care Unit, The Second Affiliated Hospital of Zhejiang University School of Medicine, Hangzhou, China

**Keywords:** extracorporeal membrane oxygenation, endovascular procedures, vascular closure devices, complications, endovascular aortic repair

## Abstract

**Background:** Total percutaneous closure for the site of femoral arterial puncture using Perclose ProGlide (PP) has become prevalent post-percutaneous endovascular aortic repair (EVAR) and veno-arterial extracorporeal membrane oxygenation (VA-ECMO).

**Objective:** To evaluate the safety and efficacy of total percutaneous closure of the femoral artery access site post-EVAR compared with VA-ECMO.

**Methods:** This was a retrospective observational study conducted over 4 years, including 88 patients who underwent EVAR (64 patients) and VA-ECMO (24 patients). Perclose ProGlide devices were used in the femoral artery puncture sites closed percutaneously. In this study, technical success was defined as successful arterial closure of the common femoral artery (CFA) without additional surgical or endovascular procedures to prevent vessel leaking. Access site complications, including overt bleeding requiring transfusion or surgical intervention, minor bleeding, tinea cruris, pseudoaneurysm, and lymphocele, were recorded 24 h and 30 days after arterial closure.

**Results:** Each group's technical success rates were 95.8% (VA-ECMO) and 92.2% EVAR, respectively. There were no differences in the periprocedural complications of major bleeding, pseudoaneurysm, minor bleeding, acute limb ischemia, and groin infection. Furthermore, we did not observe any complications such as arterial thrombosis, dissection, stenosis, arteriovenous fistula, hematoma, groin infection, or lymphocele at the access site by following-up an ultrasound examination. There was no significant difference in the technical success rate of percutaneous closure by the PP device in the EVAR and VA-ECMO oxygenation groups. Also, no periprocedural or 30-day complications were observed at the access site of the EVAR and VA-ECMO patients.

## Introduction

Recent studies revealed that Veno-arterial extracorporeal membrane oxygenation (VA-ECMO) as a kind of mechanical circulatory and gas exchange support could benefit patients with shock or without return of spontaneous circulation during cardiorespiratory resuscitation (1–3). Moreover, endovascular aortic repair (EVAR) has spread rapidly as an alternative to treat abdominal aortic aneurysms (4–8). The most frequently accessed site for VA-ECMO is through the common femoral artery (CFA), using either open or percutaneous techniques. Percutaneous closure devices for a femoral arterial access site have been approved for use with up to only 10 French (Fr) sheaths in the past decades (9, 10). However, recently, the Perclose ProGlide (PP) suture-mediated closure technique (Abbott Laboratories, Chicago, IL, USA) has made it possible to close vessels in which larger sheaths are required (11, 12).

The PP percutaneous technique has been extensively used in endovascular therapy. Torsello et al.'s prospective randomized study indicates that compared with traditional surgical cutdown, the PP percutaneous technique showed several benefits, including a lower complication rate at the percutaneous group access site (5). interestingly, total percutaneous closure of CFA access sites highly increases patient comfort while decreases wound infection and lymph fistula rate dramatically (13, 14). Patients are also mobilized and discharged earlier following the use of percutaneous closure devices than compression (15, 16), which implies its promising prospect.

Although studies of the complication and success rates of percutaneous closure devices have accumulated in the past two decades (5, 6, 8, 9, 11, 13, 17–20), there is no data on applying the PP technique VA-ECMO patients. Furthermore, there are no comparisons of the PP method between VA-ECMO and percutaneous EVAR patients. Thus, our study aimed to compare the success rates and complications of the PP suture-mediated closure technique. In patients with these two different pathophysiological conditions.

## Materials and Methods

Patients who received total percutaneous closure of a femoral access site to wean VA-ECMO or finish EVAR procedures, between February 2015 and October 2018, in The Second Affiliated Hospital of Zhejiang University School of Medicine, Hangzhou, China, were examined retrospectively. The puncture sites of all patients were evaluated by ultrasound before the procedure. All patients' demographic characteristics, comorbidities, and routine biochemical analyses were utterly documented. At The Second Affiliated Hospital, the ethics committee, Zhejiang University, approved our study protocol.

Each PP closure device was inserted by the same trained and experienced operators into the anesthetized patient. Femoral artery ultrasound was used for the vessel diameter and calcification measurement before the placement of the PP closure device. A small skin incision was made to permit the advancement and deployment of the PP device over a 0.035-inch guidewire. On the VA-ECMO withdrawal day and at the end of EVAR, the arterial sheath was removed, leaving a guidewire in the artery. Two sutures were placed in each arteriotomy using either two 8-Fr PP closure devices sequentially deployed with opposite 30° rotation in a “crosshair” configuration. While one operator manually compressed the puncture site, the other operator tightened the knot with the knot pusher. A third PP device could be applied if necessary. After achieving hemostasis, the guidewire was quickly removed, and additional manual compression was applied as needed for oozing bleeding.

Procedural success was defined as successful arterial closure of the CFA without additional surgical or endovascular procedures to prevent vessel leaking. The Bleeding Academic Research Consortium (BARC) highly suggested using the bleeding classification measurement in our research (21). Access-related complications including periprocedural hemorrhoid, acute hindlimb ischemia, tinea cruris, multiple system/organ failure, femoral arterial stenosis, arterial thrombosis and dissection, pseudoaneurysm, arteriovenous fistula, hematoma, or lymphocele in the following 30 days post-arterial closure of CFA.

Continuous variables were given as mean ± standard deviation (SD) or median (interquartile) for skewed variables, while categorical data were expressed as number and percent as we previously described. The statistical difference for continuous variables was based on the Kolmogorov–Smirnov test, whereas categorical variables were assessed using a chi-square test or Fisher's exact test, as appropriate. The Student's *t*-test or Mann–Whitney U-test was used for comparing the groups' continuous variables according to whether or not they were normally distributed. Results were evaluated within a 95% confidence interval and at a significance level of *p* < 0.05. All statistical analyses were performed using SPSS (version 11.0).

## Results

A total of 88 patients, including 24 patients who underwent VA-ECMO and 64 patients who received EVAR treatment, were included in this study. Demographic characteristics and current comorbidities of the patients in VA-ECMO and EVAR subgroups who received PP closure treatment were analyzed. Characteristics at the inception of the study are presented in Table 1. The VA-ECMO patients were significantly younger than the EVAR patients. There was no significant difference between the two groups regarding body mass index (BMI), diabetes mellitus, and coronary artery disease (CAD). Compared with the VA-ECMO patients, the EVAR patients were associated with higher hypertension, hyperlipidemia, and smoking. However, compared with the EVAR patients, the VA-ECMO patients were associated with higher heart and respiratory failure incidences.

**Table 1:** Characteristics of patients undergoing VA-ECMO and EVAR.

**Characteristic**	**VA-ECMO**	**EVAR**	***P***
	**(*n* = 24)**	**(*n* = 64)**	
Age (years)	42.0 ± 19.5	68.7 ± 10.9	<0.001
Male sex (*n*, %)	10 (47.6%)	51 (79.7%)	0.010
Body mass index (BMI) (kg/m^2^)	24.3 ± 3.8	25.6 ± 4.8	0.262
Hypertension (*n*, %)	4 (19.0%)	61 (95.3%)	<0.001
Diabetes mellitus (*n*, %)	2 (9.5%)	4 (6.3%)	0.634
CAD (*n*, %)	1 (4.8%)	16 (25%)	0.059
Hyperlipidemia (*n*, %)	2 (9.5%)	41 (64.1%)	<0.001
Heart failure (*n*, %)	20 (95.2)	2 (3.1%)	<0.001
Respiratory failure (*n*, %)	8 (38.1%)	1 (1.6%)	<0.001
Antiplatelet (*n*, %)	6 (28.6%)	9 (14.1%)	0.185
Smoking (*n*, %)	3 (14.3%)	45 (70.3%)	<0.001

*CAD, coronary artery disease; VA-ECMO, veno-arterial extracorporeal membrane oxygenation; EVAR, endovascular aortic repair*.

Most of the CFA access procedures were performed successfully, without conversion to open surgery. However, two patients received immediate surgical intervention due to the failure of the PP closure device. The 64 patients in the EVAR group received percutaneous closure using PP devices in 128 CFAs, whereas all 24 VA-ECMO patients received unilateral CFA access and percutaneous closure (Table 2). The patients in the EVAR group were associated with a larger and more severely calcified CFA compared with the VA-ECMO patients. There was no difference in the sheath size in the two groups (Table 2). The patients' total success rates in the VA-ECMO and EVAR groups were similar (95.8 and 92.2%, respectively). Due to the device failure, 2 VA-ECMO patients and 16 patients who underwent EVAR treatment required a third PP closure device to close the access site fully. One patient in each group had complete device failure and required surgical repair because of FA pseudoaneurysm and hematoma in the vascular access site 3 days post-percutaneous closure (Table 2).

**Table 2:** Periprocedural characteristics of VA-ECMO and EVAR patients^*^.

**Characteristic**	**VA-ECMO**	**EVAR**	***p*-Values**
	***(n* = 24)**	**(*n* = 64)**	
Puncture sites (common femoral arteries)	24	128	
Hospital stay (days)	13.7 ± 10.9	9.2 ± 6.9	0.024
Blood transfusion, % (*n*/*N*)	4/24 (16.7%)	4/64 (6.3%)	<0.001
Periprocedure anticoagulation, % (*n*/*N*)	19/24 (79.2%)	60/64 (93.8%)	0.058
Access site			
CFA diameter (mm)	6.9 ± 0.7	7.1 ± 0.8	0.283
vCFA calcification, % (*n*/*N*)	2/24 (8.3%)	49/64 (76.6%)	<0.001
Sheath size			
<18 Fr, % (*n*/*N*)	6/24 (25.0%)	28/128 (21.9%)	0.791
Technique success rate, % (*n*/*N*)	23/24 (95.8%)	118/128 (92.2%)	0.999
Device failure			
Primary device failure	2/24 (8.3%)	16/128 (12.5%)	0.999
Complete device failure	1/24 (4.2%)	1/128 (0.8%)	0.292
No. of Perclose ProGlide	2.1 ± 0.3	4.3 ± 0.5	<0.001

* *CFA, common femoral artery; VA-ECMO, veno-arterial extracorporeal membrane oxygenation; EVAR, endovascular aortic repair*.

There were no differences in the periprocedural complications of major bleeding, pseudoaneurysm, minor bleeding, acute limb ischemia, and groin infection. We did not observe any stenosis, arterial thrombosis, and dissection, pseudoaneurysm, arteriovenous fistula, hematoma, groin infection, or lymphocele by ultrasound test in the access site (Table 3).

**Table 3:** Periprocedural and 30-day complications of VA-ECMO and EVAR patients.

**Complications**	**VA-ECMO**	**EVAR**	***p-*Values**
**(1) 24-h Vascular Access Complications**			
**Major complication**			
Major bleeding •(intervention or •transfusion acquired)	1/21 (4.8%)	6/128 (4.7%)	0.999
Peudoaneurysm	1/21 (4.8%)	4/128 (3.1%)	0.537
**Minor complication**			
Minor bleeding	3/21 (14.3%)	18/128 (12.3%)	0.731
Peudoaneurysm	1/21 (4.8%)	4/128 (3.1%)	0.537
Acute lower limb ischemia (acute arterial dissection/occlusion)	0/21	2/128 (1.6%)	0.999
Groin infection	0/21	2/128 (1.6%)	0.999
**(2) 30-Day Vascular Access Complications**			
Arterial thrombosis	0/21	2/128 (1.6%)	0.999
Arterial dissection	0/21	0/128	0.999
Pseudoaneurysm	1/21 (4.8%)	4/128 (3.1%)	0.527
Stenosis (>50%)	0/21	2/128 (1.6%)	0.999
Arteriovenous fistula	0/21	0/128	0.999
Hematoma	1/21 (4.8%)	6/128 (4.7%)	0.999
Groin infection	0/21	2/128 (1.6%)	0.999
Lymphocele	0/21	1/128 (0.8%)	0.999

*VA-ECMO, veno-arterial extracorporeal membrane oxygenation; EVAR, endovascular aneurysm repair*.

## Discussion

We revealed that the incidence of PP closure device-related complications and the device technique success rate were similar in EVAR and VA-ECMO patients. The technical success rates of percutaneous closure of vascular access sites in VA-ECMO and EVAR patients are 95.8 and 92.2%, respectively. In all, our research indicated that the necessity of intraoperative and post-operative transfusion is similar in both groups.

The PP closure device system was the first suture-mediated device approved by the United States Food and Drug Administration. Since then, the development of the PP closure device has evolved (22). As the latest generation, the suture-mediated device from Abbot, PP closure device offers a breakthrough in the ease of knot delivery, trimming of the suture, and polypropylene monofilaments sutures, which are non-inflammatory and characterized by higher tensile strength (22). The deployment of the PP device includes several steps that require meticulous care and are prone to failure if operators are not adequately trained. Dr. Balzer et al.'s research uncovered that the learning curve of suture-based closure device's technical success was steeper and much more enduring than traditional methods (23).

Over the past few decades, EVAR has become the preferred treatment choice for patients with an anatomically suitable abdominal aortic aneurysm (24). Total percutaneous EVAR minimizes invasiveness compared with femoral cutdown access EVAR. Several small single-center studies using various grafts show a reduction in total operative time and hospital stay length (4, 5, 10, 11, 25, 26). Previous studies have reported that vascular access site complications range from 0 to 11% (4, 13). Thus, percutaneous EVAR has been shown to have a higher success rate, shorter operation time, shorter length of hospital stay, and fewer access site complications than cutdown EVAR. Similarly, total percutaneous peripheral VA-ECMO minimizes invasiveness compared with femoral cutdown VA-ECMO with the femoral artery access. Peripheral VA-ECMO remains one of the most widely used and reliable methods as acknowledged for rescuing perfusion in life-threatening circulatory and respiratory failure (27). This extracorporeal support strategy provides immediate restitution of organ perfusion and oxygenation and, therefore, enables clinicians to establish a bridge to decision, recovery, or alternative therapies in various settings. However, there were limited data on the percutaneous closure of the vascular access sites by the PP closure device in VA-ECMO patients. Data from this study demonstrate that the meticulous use by a well-trained surgeon of two PP devices for percutaneous closure of a femoral artery access site in VA-ECMO patients is safe and effective compared to total percutaneous EVAR.

Four VA-ECMO and 24 EVAR patients did, however, have some closure site bleeding. Most of these incidences could be managed by manual compression. Only five patients needed further surgical intervention to stop the bleeding. We found that these five patients, who needed a transfusion and surgical intervention, had severe femoral artery calcification. Fortunately, it was evident that pulsatile bleeding was from the puncture site when the PP device technique failed. Maintaining stiff guidewire access until confirmation of adequate hemostasis is critically important (11, 12, 28), especially in VA-ECMO patients. In cases of PP device failure, the guidewire allows the bleeding to be wholly and immediately stopped with a dilator's simple reinsertion to prevent a life-threatening hemorrhagic complication. This guide wire advantage allows enough time for an unhurried surgical repair of the femoral artery access site to be performed.

In contrast, there were two acute limb ischemic failures in EVAR patients due to an anterior plaque that had fractured and resulted in local dissection occluding the distal flow. These patients received surgical intervention for revascularization before the irreversible injury of the limb. Based on our experience, cannulation of profunda, or superficial FA could be one of the major causes of vessel rupture or occlusion, especially when the arterial puncture site is too low. These complications can be avoided by the identification of the CFA by ultrasound. For example, we detected 50% CFA stenosis by ultrasound in two EVAR patients who did not show obvious limb ischemic symptoms at the 30-day follow-up. However, the ultrasound revealed a posterior plaque fracture that resulted in a local dissection and thrombosis that occluded the distal flow. These patients received a third PP device that was deployed due to primary device failure during the procedure.

There were four pseudoaneurysms in the EVAR patients and one pseudoaneurysm in the VA-ECMO patients. Three of the pseudoaneurysms were related to closure device failure, whereas two were due to mycotic aneurysm, diabetes, and groin infection. Regardless of the pseudoaneurysms' etiology, they all required major arterial reconstructions and were characterized by significant morbidity. These complications emphasized the importance of maintaining strict aseptic techniques and anti-infective therapy in the perioperative period.

Our study design was a retrospective, non-randomized, and observational study with a relatively small number of patients at a single center. Because of the lack of randomization, the surgeon's preference and experience likely played a role in treatment choice. Besides, the database did not provide information on long-term follow-up and prevented us from comparing the incidence of iliofemoral stenosis. Prospective, randomized clinical trials should confirm our findings with a larger population.

In conclusion, this study demonstrated that two PPs for percutaneous closure of femoral artery access site in VA-ECMO and EVAR patients is a safe and effective procedure when used with a well-trained surgeon and careful patient selection. The technical success rate and device-related complications are similar in the EVAR and VA-ECMO patients. Long-term follow-up is still necessary.

## Data Availability Statement

The raw data supporting the conclusions of this article will be made available by the authors, without undue reservation.

## Ethics Statement

The studies involving human participants were reviewed and approved by Human Research Ethics Committee of the second affiliated hospital of Zhejiang University School of Medicine. The patients/participants provided their written informed consent to participate in this study.

## Author Contributions

ZLiu, MH, BC, and YX: conception and design and final approval of the article. ZLiu, YX, MH, CS, XX, YP, LY, ZL, PH, and LZ: analysis and interpretation. ZLiu, YX, MH, CS, XX, ZL, PH, and LZ: data collection. ZL, YX, and LY: writing the article and statistical analysis. ZLiu, YX, MH, CS, XX, YP, LY, ZL, PH, LZ, MH, and BC: critical revision of the article. ZLiu and LY: obtained funding. ZLiu, MH, and BC: overall responsibility. All authors contributed to the article and approved the submitted version.

## Conflict of Interest

The authors declare that the research was conducted in the absence of any commercial or financial relationships that could be construed as a potential conflict of interest.

## Publisher's Note

All claims expressed in this article are solely those of the authors and do not necessarily represent those of their affiliated organizations, or those of the publisher, the editors and the reviewers. Any product that may be evaluated in this article, or claim that may be made by its manufacturer, is not guaranteed or endorsed by the publisher.
